# Pharmacological mechanisms of probenecid for SARS-CoV-2 and RSV co-infection: findings of system pharmacology, molecular docking, molecular dynamics simulation, and structure–activity relationship

**DOI:** 10.3389/fmicb.2025.1552603

**Published:** 2025-04-30

**Authors:** Junbin Hong, Zhendong Guo, XiaoMei Huang, Peng Wu, Xinying Chen, Xiaoyi Liu, Jinghua Yang, Yanni Lai

**Affiliations:** ^1^Guangzhou University of Chinese Medicine, Guangzhou, China; ^2^School of Medicine and Health, Shunde Polytechnic, Foshan, China

**Keywords:** probenecid, SARS-CoV-2, RSV, co-infection, system pharmacology, molecular docking, molecular dynamics simulation, structure-activity relationship

## Abstract

**Background:**

The clinical consequences of the co-infection with novel severe acute respiratory syndrome coronavirus 2 (SARS-CoV-2) and respiratory syncytial virus (RSV) are not optimistic. Nevertheless, there is currently no approved therapeutic regimen specifically targeting SARS-CoV-2/RSV co-infection, with existing monotherapies showing limited efficacy. According to recent studies, probenecid has both anti-SARS-CoV-2 and anti-RSV effects. Therefore, as one probable molecular candidate for the co-infection with SARS-CoV-2 and RSV, probenecid was researched in this exploration.

**Methods:**

Using systems pharmacology and bioinformatics, we characterized the targets associated with probenecid for the treatment of SARS-CoV-2/RSV co-infection, focusing on their biological functions, mechanisms and binding activities. To further validate these findings, we conducted molecular docking, MD simulations, electrostatic potential mapping, and SAR analysis to explore the binding interactions between probenecid and the identified core targets.

**Results:**

We identified 141 targets that overlapped with the co-infection and probenecid, and used these shared targets to construct a protein-protein interaction (PPI) network. Subsequently, we obtained the top 16 hub targets of probenecid for SARS-CoV-2/RSV co-infection, namely, AKT1, ALB, EGFR, CASP3, CTNNB1, SRC, HSP90AA1, and so on. According to the enrichment analysis, probenecid might affect inflammation, immunity, oxidative stress, and virus defenses; Toll-like receptor, TNF, IL-17, NOD-like receptor, cytokine-cytokine receptor, among others. Additionally, based on molecular docking analysis, probenecid is effectively bound to the targets related to the SARS-CoV-2/RSV co-infection. Meanwhile, according to molecular dynamics (MD) simulations and structure-activity relationship (SAR) analysis, we speculated that SRC and HSP90AA1 are more likely to be the target proteins of probenecid than the other proteins.

**Conclusion:**

Our findings from systems pharmacology and bioinformatics analysis indicate that immune and inflammatory responses play a pivotal role in the therapeutic effects of probenecid. Infectious disease-related pathways also contribute significantly to its effectiveness in treating SARS-CoV-2/RSV co-infection. Further validation was conducted through molecular docking, MD simulations, electrostatic potential mapping, and SAR analysis. These analyses suggest that SRC and HSP90AA1 are the potential binding targets of probenecid. This study provides valuable preliminary insights into the molecular mechanisms of probenecid. It establishes a strong foundation for future research to explore its potential as a therapeutic strategy for SARS-CoV-2/RSV co-infection.

## Introduction

Coronavirus disease 2019 (COVID-19) caused by severe acute respiratory syndrome coronavirus 2 (SARS-CoV-2), emerged and disseminated worldwide in December 2019 (Wiersinga et al., 2020). Afterward, it was declared a pandemic by the World Health Organization (WHO) in March 2020 and contributed to 777,637,340 confirmed cases, including 7,090,480 deaths^1^ to date. Due to its high pathogenicity and mortality, COVID-19 poses a challenge to research in human medicine, medical care, and the prevention and control of the diseases worldwide (Liang et al., 2020).

Similarly, RSV is the main causative agent of acute respiratory tract infections (ARTIs) that primarily affects pediatric, elderly, and immunocompromised populations with over 33 million annual cases worldwide (Langedijk and Bont, 2023). Moreover, it has propagated dramatically in some countries (Cong et al., 2024; Li Y. et al., 2022) due to the relaxation of COVID-19 restrictions (Eden et al., 2022; Kuitunen et al., 2022; Nygaard et al., 2023) and other factors. An increased incidence of these two epidemics is alarming as the co-infection of respiratory syncytial virus (RSV) and SARS-CoV-2 may further increase morbidity and mortality (Tripp and Martin, 2022). Specifically, throughout the SARS-CoV-2 pandemic, co-infections of SARS-CoV-2 and RSV have been frequently documented in clinical reports (Deval et al., 2024; Trifonova et al., 2022; Wu et al., 2025). According to a recent study, the incidence of SARS-CoV-2/RSV co-infection significantly increased during the Omicron variant wave, and co-infection patterns may be influenced by viral variants (Tai et al., 2025). In populations in Northern California, recurrent cases of SARS-CoV-2/RSV co-infection have been observed. Among 116 SARS-CoV-2-positive specimens, over 20% contained at least one additional respiratory pathogen, including RSV (Kim et al., 2020). Moreover, according to a study conducted in the United States, 15.8% of the children diagnosed with an infection with SARS-CoV-2 were also co-infected with other viruses, among which 66.8% were RSV (Wanga et al., 2021). In addition, children in hospitals experienced more severe disease when SARS-CoV-2 and RSV co-infected (Agathis et al., 2023; Cong et al., 2022). Reportedly, SARS-CoV-2/RSV co-infection attributed to a surged proportion of infantile COVID-19 that required oxygen therapy for severe bronchiolitis between 2019 and 2020 (Rotulo et al., 2021). Therefore, the potential association between SARS-CoV-2 and RSV co-infection needs an in-depth investigation. Although SARS-CoV-2 vaccines and several antiviral drugs (e.g., Nirmatrelvir) have been developed and are widely used (Senevirathne et al., 2024), emerging resistance to Nirmatrelvir poses a challenge to clinical efficacy (Duan et al., 2023). Similarly, vaccines against RSV have been introduced for the elderly population; however, vaccines are unavailable for children and immunocompromised patients with inadequate treatment strategies (Zhang et al., 2023). In a briefing released in December 2024, the FDA noted that Moderna’s mRNA RSV vaccine candidates (mRNA-1345 and mRNA-1365) posed a risk of symptom worsening in infants and young children, leading to the suspension of all pediatric RSV vaccine clinical trials (Mahase, 2024). To mitigate the impact of co-infected diseases on healthcare systems worldwide, extensive medical research is crucial for identifying potential therapeutic targets for SARS-CoV-2/RSV co-infection.

Probenecid is a clinically safe and readily available drug used for the treatment of gout (Onodi et al., 2023). It affects ion channels such as adenosine-triphosphate (ATP) release channel pannexin 1 and has anti-inflammatory effects (Cicero et al., 2021). Interestingly, recent studies have shown that treatment with probenecid is effective in reducing RSV and SARS-CoV-2 viral replication (Murray et al., 2022; Tripp and Martin, 2022). Additionally, in a Phase II clinical trial involving non-hospitalized patients with mild to moderate COVID-19, probenecid demonstrated significant clinical efficacy: the median time to viral clearance was significantly reduced to 7 days in the high-dose group, and the complete symptom resolution rate on day 10 of treatment was significantly higher, with a favorable safety profile (Martin et al., 2023). The researchers speculated that the solute carriers (SLCs) and their roles as organic anion transporters (OATs) may be connected to the antiviral mechanism of action. SARS-CoV-2 exploits angiotensin-converting enzyme 2 (ACE2) as its primary receptor to initiate host cell invasion. Additionally, Pannexin-1 (PANX-1) is pathologically hyperactivated during SARS-CoV-2 infection, driving excessive release of pro-inflammatory mediators (ATP, PGE2, IL-1β) (Luu et al., 2021), suggesting its potential as a therapeutic target. Studies demonstrate that probenecid exhibits dual antiviral and anti-inflammatory effects through coordinated modulation of ACE2 and PANX1 pathways. Probenecid downregulates ACE2 expression (Sinha et al., 2020), reducing the availability of spike protein-binding sites on host cell surfaces, thereby limiting SARS-CoV-2 entry. Concurrently, probenecid directly inhibits PANX1 channel activity (Swayne et al., 2020), effectively blocking the inflammatory cascade triggered by SARS-CoV-2 infection. Additionally, probenecid inhibits RSV replication in human respiratory epithelial cells by suppressing the MAPK pathway (via blocking RSV-induced JNK/ERK phosphorylation), which disrupts AP-1 transcription complex formation (through c-jun inhibition) and reverses HNF-4 repression, thereby providing a dual mechanism to impede viral replication (Jones et al., 2024). Consequently, we hypothesized that probenecid could be tested for treating SARS-Cov-2/RSV co-infection.

**FIGURE 1:**
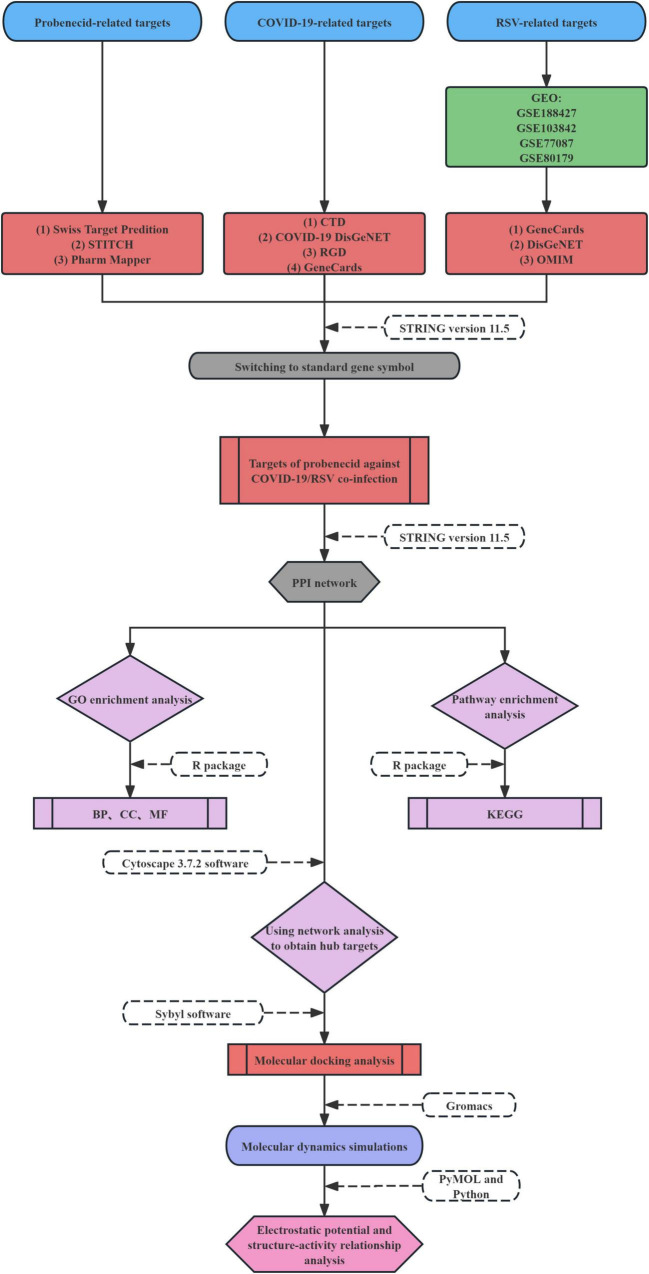
Overall workflow of the present study.

The host-based targets and molecular mechanisms of probenecid in SARS-CoV-2/RSV co-infection remain unclear. In this study, we utilized relevant bio-datasets and web databases to investigate the potential mechanism of action of probenecid against SARS-CoV-2/RSV co-infection. Additionally, we employed systems pharmacology, molecular docking, molecular dynamics (MD) simulation, electrostatic potential analysis, and structure-activity relationship (SAR) analysis to explore the target genes and signaling pathways involved in the anti-co-infection effects of probenecid. The workflow for this investigation is illustrated in Figure 1.

## Materials and methods

### Screening of probenecid-related targets

Probenecid-related pharmacological targets were collected from the following databases: (1) Swiss Target Prediction^2^ (Daina et al., 2019), (2) Chemical Association Networks (STITCH)^3^ (Szklarczyk et al., 2016), and (3) Pharm Mapper^4^ (Wang et al., 2017). To standardize the target protein names, the STRING database (version 11.5)^5^ (Szklarczyk et al., 2019) was used with the organism restricted to “Homo sapiens.”

### Identification of SARS-CoV-2/RSV co-infection-associated genes

The following four open-source databases were employed to collect COVID-19 associated targets: (1) CTD^6^ (Davis et al., 2021), (2) COVID-19 DisGeNET data collection^7^ (Pinero et al., 2020), (3) RGD Disease Portals^8^ (Smith et al., 2020), and (4) GeneCards^9^ (Stelzer et al., 2016).

In addition, RSV infection-related target genes were determined by the differentially expressed genes (DEGs) from the GEO database (Barrett et al., 2013) (GSE188427, GSE103842, GSE77087, and GSE80179 datasets) and were analyzed using GEO2R. To identify DEGs from datasets, the Benjamini-Hochberg adjusted *p*-value (False discovery rate, FDR) < 0.05 and | log2FC| ≥ 1 criteria were used. Moreover, RSV-related human target genes were screened through the following three databases: (1) GeneCards (see text footnote 9) (Stelzer et al., 2016), (2) DisGeNET^10^ (Pinero et al., 2020), and (3) Online Mendelian Inheritance in Man (OMIM)^11^ (Amberger et al., 2015).

### Targets of probenecid against SARS-CoV-2/RSV co-infection acquisition

The targets of probenecid against SARS-CoV-2/RSV co-infection were identified at the intersection of probenecid-related and SARS-CoV-2/RSV co-infection-related targets. To determine the shared targets, the jvenn tool^12^ (Bardou et al., 2014) was utilized.

### Analysis of protein-protein interaction and network structure

Furthermore, the protein-protein interaction (PPI) network was constructed using STRING, with a minimum interaction score of 0.4 and the organism set to “Homo sapiens” to identify the hub targets. The resulting PPI network was visualized and analyzed using Cytoscape 3.8.2, and the degree values of the targets were calculated using the NetworkAnalyzer plugin. Targets with degree values greater than twice the mean were selected as hub targets. The PPI network helps to understand the biological processes underlying the pathogenesis of these targets at the protein level.

### Gene ontology and pathway enrichment analysis

Gene ontology (GO) analysis, including molecular function (MF), cellular component (CC), and biological process (BP), as well as Kyoto Encyclopedia of Genes and Genomes (KEGG) pathway enrichment analysis, were conducted using the R package “clusterProfiler” to explore the biological mechanisms and signaling pathways of shared host factor networks. An adjusted *P*-value < 0.05 was used to filter significantly enriched terms, and the “ggplot2” package in R was used to visualize the top 8 GO terms and 30 KEGG pathway terms.

### Molecular docking analysis

Molecular docking was performed using the Surflex docking module in Sybyl-X2.1.1 to predict the binding models between probenecid and the top 10 hub targets with the highest degree values. The ligand from the same active pocket of the protein was used as a positive control. The molecular structures of probenecid and the targets were obtained from the PubChem and RCSB Protein Data Bank (PDB) database^13^ (Sussman et al., 1998), respectively. In addition, the receptor-ligand interaction was visualized via Discovery Studio 2016 software by selecting seme-flexible docking with an expansion coefficient of 1, threshold value of 0.5, and system default value as docking parameters. The total score was employed to evaluate the docking situation, which is in inverse proportion to the binding steadiness of the composite (Li et al., 2013). Total scores of > 3, ≥ 6, and ≥ 9 indicated the existence, good, and strong docking activity between the probenecid and the top 10 hub-targets, respectively (Todeschini et al., 2016).

### MD simulations

MD simulations were conducted using the CUDA-accelerated Gromacs2022.2 program with the Charmm36 force field (Huang and MacKerell, 2013; Van Der Spoel et al., 2005) to obtain the complex structures of AKT serine/threonine kinase 1 (AKT1), epidermal growth factor receptor (EGFR), SRC proto-oncogene, non-receptor tyrosine kinase (SRC), heat shock protein 90 alpha family class A member 1 (HSP90AA1), and peroxisome proliferator activated receptor gamma (PPARG) protein proteins bound to the probenecid ligand. These complexes were generated through molecular docking and used as the starting coordinate files. The complexes were solvated in a cubic periodic boundary box using the TIP3 water model, and the system charges were neutralized by adding sodium chloride to a concentration of 0.15 mol/L. Energy minimization was performed using the steepest descent method to remove unfavorable contacts within the system. Subsequently, canonical (NVT) and isothermal-isobaric (NPT) ensembles were used for 100 ns MD simulations, with the system temperature maintained at 310 K and pressure at 1 bar.

### Electrostatic potential and structure-activity relationship analysis

The optimized probenecid-protein complex conformations were imported into PyMOL to generate electrostatic potential maps, enabling a systematic analysis of the binding interactions and potential binding patterns between the small molecule and the target protein. These electrostatic surface maps provide an intuitive visualization of the protein’s electrostatic distribution, helping to identify key binding regions and interaction types between the ligand and the protein. Additionally, after removing periodic boundary conditions (PBC) from the MD) trajectory, dynamic interaction profiles were generated using Python (version 3.9) and the ProLif package (version 2.5.4). These profiles illustrate the dynamic interaction patterns between the ligand and protein throughout the simulation, including the types and duration of hydrogen bonds, van der Waals forces, and hydrophobic interactions.

## Results

### Identification of 141 common genes associated with SARS-CoV-2/RSV co-infection and probenecid

**FIGURE 2:**
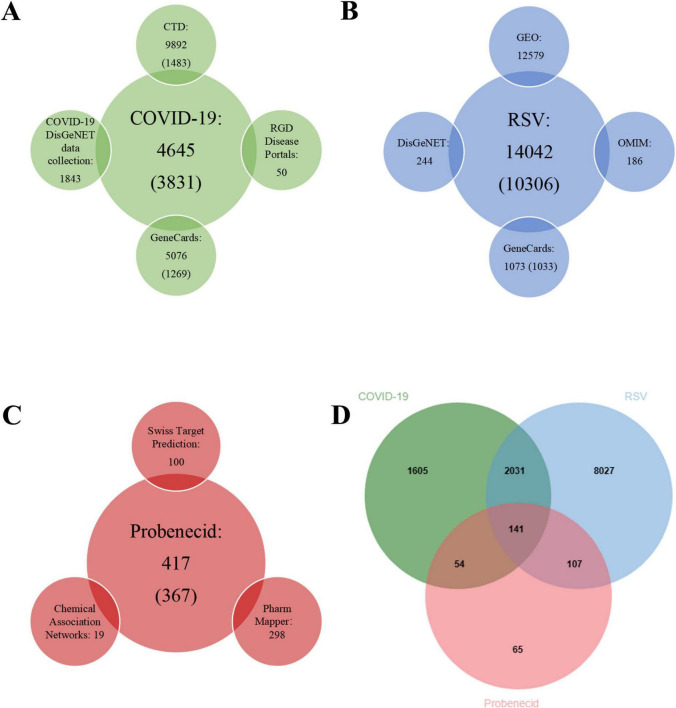
The number of related target genes, databases, and Venn diagram. **(A)** The number of COVID-19-related target genes from four open-source databases. **(B)** The number of RSV-related target genes from four open-source databases. **(C)** The number of probenecid-related target genes from three open-source databases. **(D)** Venn diagram depicting shared target genes between COVID-19, RSV, and probenecid.

Figure 2 illustrates the process of identifying common targets for SARS-CoV-2/RSV co-infection and probenecid. To screen for probenecid-related targets, we utilized three databases: Swiss Target Prediction (100 targets), Chemical Association Networks (STITCH) (19 targets), and PharmMapper (298 targets). After removing duplicates and standardizing gene symbols, a total of 367 unique targets were identified (Figure 2A).

Next, we retrieved COVID-19-associated target genes from four public databases: GeneCards (5,076 targets), the COVID-19 DisGeNET data collection (1,843 targets), RGD Disease Portals (50 targets), and CTD (9,892 targets). Since the number of targets from CTD and GeneCards was excessively large, we empirically selected the top 15% of CTD targets (1,483 targets) and the top 25% of GeneCards targets (1,269 targets) based on inference and relevance scores. After eliminating duplicates, a total of 3,831 COVID-19-associated genes were identified (Figure 2B).

**FIGURE 3:**
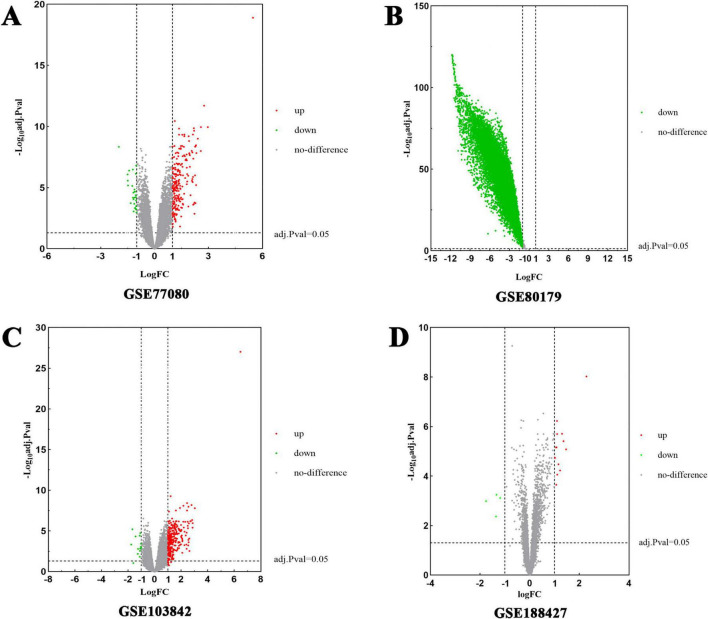
Volcano plots of differentially expressed genes (DEGs) for RSV-infected patients. The abscissa represented log FC and the ordinate indicated the –log10 (adjusted *p*-value) of the genes. The red dots on the left and the green on the right respectively indicate down- and up-regulated genes with the criteria of adjusted *p*-value < 0.05 and | log2FC| > 1. **(A)** DEGs from GSE77087 were collected from 81 RSV-infected children and 23 healthy age-matched controls. **(B)** DEGs from GSE80179 were obtained from 27 RSV-infected children and 52 healthy controls. **(C)** DEGs from GSE103842 originated from 62 infants with RSV LRTI and 12 healthy asymptomatic age-matched controls. **(D)** DEGs from GSE188427 were obtained from 147 RSV-infected children and 51 healthy controls.

Subsequently, DEGs associated with RSV infection were obtained from GEO by analyzing four datasets: GSE77087 (206 targets), GSE80179 (12,062 targets), GSE103842 (294 targets), and GSE188427 (17 targets) datasets. The GSE77087 dataset contained whole blood gene expression profiles from 81 RSV-infected children and 23 age-matched healthy controls, while GSE80179 included data from 27 RSV-infected children and 52 healthy controls. The GSE103842 dataset provided whole blood transcriptional profiles from 62 infants with RSV-induced lower respiratory tract infections (LRTI) and 12 healthy, asymptomatic age-matched controls. Similarly, GSE188427 contained transcriptional data from 147 RSV-infected children and 51 healthy controls. Figures 3A–D present the volcano plots depicting dysregulated DEGs in patients with RSV infection. Additionally, RSV-related target genes were identified from three databases: GeneCards (1,073 targets), DisGeNET (244 targets), and OMIM (186 targets). From GeneCards, 1,033 targets with a relevance score of ≥ 1 were retained. After removing duplicates, a total of 10,306 unique target genes were identified (Figure 2C).

Finally, 141 common genes related to SARS-CoV-2/RSV co-infection and probenecid were identified using the jvenn tool (Figure 2D). These genes were then analyzed to determine the key hub targets in the PPI network, providing insights into the potential mechanisms of probenecid against SARS-CoV-2/RSV co-infection.

### Identification of hub targets and functional modules in the PPI network of probenecid against SARS-CoV-2/RSV co-infection

**FIGURE 4:**
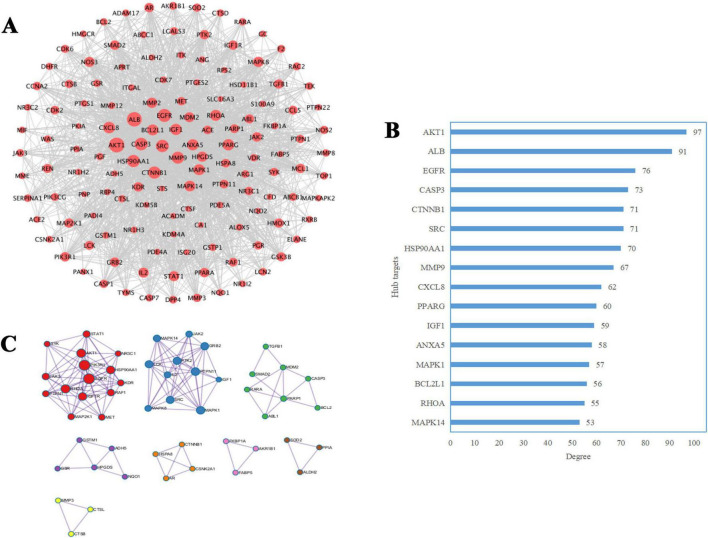
Identification of hub targets and functional modules in the protein-protein interaction (PPI) network of probenecid against SARS-CoV-2/RSV co-infection. **(A)** The red nodes represent shared therapeutic targets of probenecid against SARS-CoV-2/RSV co-infection. The node size corresponds to the degree centrality (i.e., the number of direct interactions between a node and other nodes in the network), with larger nodes indicating higher connectivity. **(B)** The blue bar chart illustrates the degree distribution of core targets associated with the synergistic anti-SARS-CoV-2/RSV effects of probenecid. The bar length is proportional to the degree value, where longer bars signify stronger hub roles of the targets within the network. **(C)** Cluster analysis of the network was performed using the Metascape database, identifying eight functional modules (indicated by distinct colors). Nodes within each module represent shared targets, and edges between nodes denote potential interactions. The module size and node connectivity reflect the functional relevance and hierarchical importance of these targets in the therapeutic mechanism.

The PPI network consisted of 137 nodes and 1,763 edges (Figure 4A), with an average node degree of 25.01, heterogeneity of 0.777, and centralization of 0.532. Nodes with degree values exceeding 50 (twice the mean degree) were identified as hub targets (Figure 4B). These included AKT1, albumin (ALB), epidermal growth factor receptor (EGFR), caspase 3 (CASP3), catenin beta 1 (CTNNB1), SRC proto-oncogene (SRC), heat shock protein 90 alpha family class A member 1 (HSP90AA1), matrix metallopeptidase 9 (MMP9), C-X-C motif chemokine ligand 8 (CXCL8), peroxisome proliferator-activated receptor gamma (PPARG), insulin-like growth factor 1 (IGF1), annexin A5 (ANXA5), mitogen-activated protein kinase 1 (MAPK1), BCL2-like 1 (BCL2L1), ras homolog family member A (RHOA), and mitogen-activated protein kinase 14 (MAPK14). Among these, the top 16 targets with the highest degree values were AKT1 (97), ALB (91), EGFR (76), CASP3 (73), CTNNB1 (71), SRC (71), HSP90AA1 (70), MMP9 (67), CXCL8 (62), PPARG (60), IGF1 (59), ANXA5 (58), MAPK1 (57), BCL2L1 (56), RHOA (55), and MAPK14 (53), as shown in Figure 4B. Therefore, these targets were prioritized as putative contributors to probenecid’s therapeutic effects against SARS-CoV-2/RSV co-infection. Furthermore, Metascape analysis revealed eight functionally distinct modules (Figure 4C), demonstrating critical associations with key biological processes: The red module was prominently associated with kinase activity regulation (e.g., AKT1, JAK3, MAP2K1) and transmembrane receptor protein tyrosine kinase signaling pathways (EGFR, IGF1R, MET). Concurrently, the blue module featured essential kinases involved in signaling cascades (JAK2, MAPK14, MAPK1). Tissue morphogenesis pathways were enriched in both the green module (TGFB1, SMAD2) and orange module (CTNNB1). The purple module demonstrated significant enrichment in cellular detoxification mechanisms (GSTM1, GSR, NQO1), while apoptotic signaling regulation was primarily mapped to the green module through critical mediators including CASP3, BCL2, and PARP1. Notably, collagen metabolic processes were distinctly clustered within the yellow module, characterized by MMP3, CTSL, and CTSB expression patterns, and so forth.

### GO enrichment analysis reveals key biological functions of probenecid in SARS-CoV-2/RSV co-infection

**FIGURE 5:**
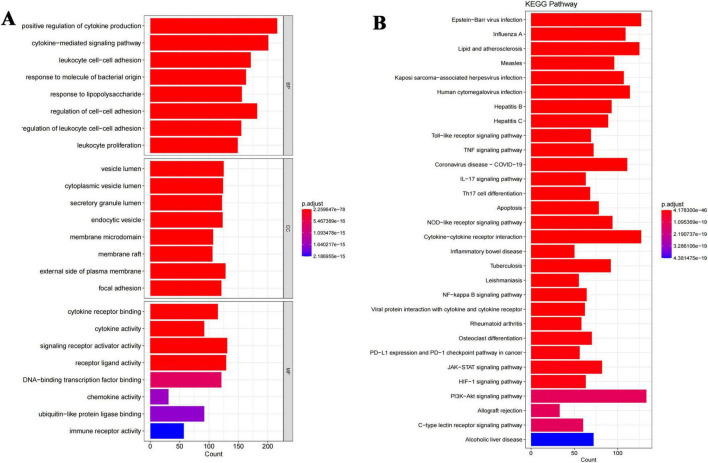
Integrated GO and KEGG enrichment analyses reveal key biological functions and pathways of probenecid against SARS-CoV-2/RSV co-infection. **(A)** Gene ontology enrichment analysis of anti-SARS-CoV-2/RSV targets, specifically including the top five biological processes (BP), cellular components (CC) and molecular functions (MF). The longer the bar, the more genes enriched; the warmer the color, the more significant the enrichment entry. **(B)** Biological pathway enrichment analysis of anti-SARS-CoV-2/RSV targets. The longer the bar, the more genes enriched; the warmer the color, the more significant the enrichment entry.

A total of 141 target genes were submitted to the R program for GO enrichment analysis to explore the biological functions potentially modulated by probenecid in SARS-CoV-2/RSV co-infection. This analysis identified 3,727 significantly enriched GO terms, including 3,262 in BP, 207 in cellular components CC, and 256 in molecular functions MF (Supplementary Table 1). As shown in Figure 5A, in the BP category, terms such as “positive regulation of cytokine production,” “cytokine-mediated signaling pathway,” and “leukocyte cell-cell adhesion” suggest that probenecid may influence inflammatory responses and immune cell interactions. The CC enrichment in terms like “vesicle lumen,” “cytoplasmic vesicle lumen,” and “secretory granule lumen” implies involvement in intracellular transport and secretion pathways that are critical during viral infections. In the MF category, the enrichment of terms such as “cytokine receptor binding,” “cytokine activity,” and “signaling receptor activator activity” may indicate that probenecid affects receptor-mediated signaling pathways linked to immune responses. These enriched GO terms provide preliminary insights into the potential biological activities of probenecid.

### KEGG enrichment analysis highlights key pathways of probenecid in SARS-CoV-2/RSV co-infection

Furthermore, KEGG enrichment analysis identified 167 crucial pathways (Supplementary Table 2) shared between probenecid and co-infection with SARS-CoV-2/RSV. The top 30 pathways, ranked by adjusted *p*-value (Figure 5B), included the Toll-like receptor signaling pathway, tumor necrosis factor (TNF) signaling pathway, Coronavirus disease-COVID-19, interleukin (IL)-17 signaling pathway, T helper cell (Th17) differentiation, and apoptosis, among others. The length and brightness of the bars in Figure 5B are proportional to the importance of each pathway. These targets were predominantly enriched in pathways related to immune and inflammatory responses, infectious diseases (especially viral infections), immune cell differentiation, and signal transduction, which aligns with the results from GO) enrichment analysis.

### Molecular docking analysis reveals strong binding of probenecid to key anti-SARS-CoV-2/RSV hub targets

**FIGURE 6:**
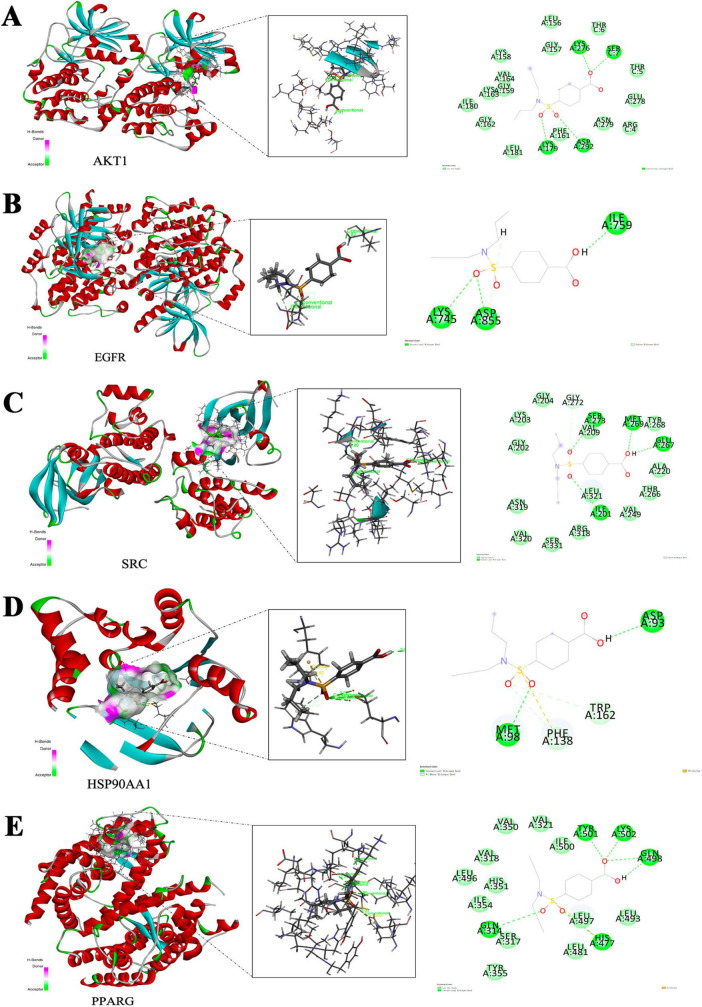
Molecular docking model of hub-targets. **(A)** AKT1. **(B)** EGFR. **(C)** SRC. **(D)** HSP90AA1. **(E)** PPARG.

The anti-SARS-CoV-2/RSV hub targets with the highest degree values (AKT1, ALB, EGFR, CASP3, CTNNB1, SRC, HSP90AA1, MMP9, CXCL8, and PPARG) were subjected to molecular docking with probenecid or their ligands. The binding stability of the protein-ligand complexes was assessed based on the total score, which reflects the binding energies of the protein-ligand complexes and their X-ray structures (Table 1). Figure 6 shows the 3D and 2D docking models of probenecid with the hub targets. The specific interactions between the hub targets and the medicine, as well as the critical residues involved in the protein-ligand complexes, were analyzed. Table 2 lists the chemical bonds associated with these residues.

**TABLE 1:** Total scores between probenecid/ligand and core proteins.

Protein name	PDB ID	Total-score	Ligand
AKT1	3ocb	6.0351	9.3306
ALB	41b2	5.2637	6.2164
EGFR	7jxq	6.5243	14.1346
CASP3	7seo	2.8349	–
CTNNB1	6o9b	5.4230	7.6536
SRC	3d7t	6.1224	8.7693
HSP90AA1	3o0i	6.7800	8.9529
MMP9	6esm	5.0123	9.0215
CXCL8	6wzm	5.0607	–
PPARG	8dsy	6.6544	9.3801

**TABLE 2:** The residues for the chemical bonds between probenecid and core proteins (≥ 6).

Protein name	Conventional hydrogen bond residue	Carbon hydrogen bond residue	Pi-donor hydrogen bond	Pi-sulfur
AKT1	LYSA276, SERC7, LYSA179, ASPA292	–	–	–
EGFR	ALYSA745, ASPA855, ILEA759	ASPA855	–	–
SRC	SERA273, METAA269, GLUA267, IL1A201	GLYA272	–	–
HSP90AA1	META98, ASPA93	-	PHEA138, TRPA162	PHEA138
PPARG	GLNA31, TYRA501, LYSA502, GLNA498, HISA477	–	–	HISA477

The docking scores of the corresponding active pocket ligands and proteins were all above 6, indicating that the docking parameters were appropriate and the results were highly reliable (Table 2). Probenecid exhibited strong binding activities with AKT1, EGFR, SRC, HSP90AA1, and PPARG, with docking scores of 6.0351, 6.5243, 6.1224, 6.7800, and 6.6544, respectively (Table 2). Additionally, probenecid formed four, three, four, two, and five conventional hydrogen bonds with AKT1, EGFR, SRC, HSP90AA1, and PPARG, respectively (Figures 6A–E). It also formed two Pi-Donor hydrogen bonds with HSP90AA1 (Table 2), and one Pi-Sulfur bond with both HSP90AA1 and PPARG (Table 2). Furthermore, various carbon-hydrogen bonds were formed with EGFR (1) and SRC (1) proteins to stabilize the complex (Table 2). These results suggest that probenecid affects the hub targets of anti-SARS-CoV-2/RSV, including AKT1, EGFR, SRC, HSP90AA1, and PPARG.

### MD simulations confirm stability of probenecid binding to SRC, HSP90AA1, and EGFR

**FIGURE 7:**
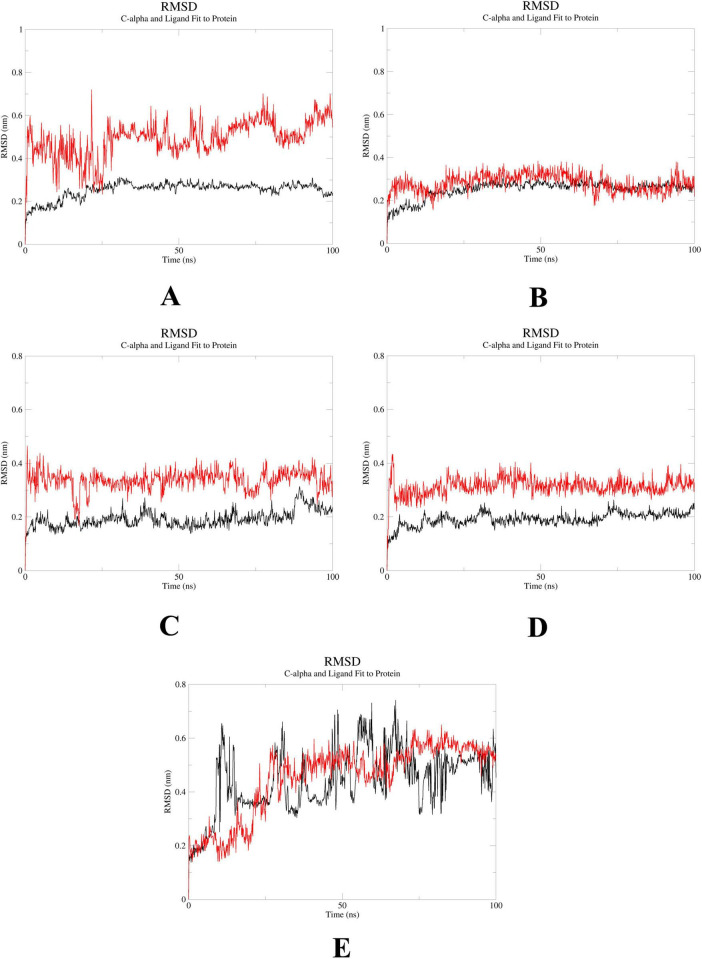
Root mean square deviation (RMSD). **(A)** System probenecid-AKT1. **(B)** System probenecid-EGFR. **(C)** System probenecid-SRC. **(D)** System probenecid-HSP90AA1. **(E)** System probenecid-PPARG.

**FIGURE 8:**
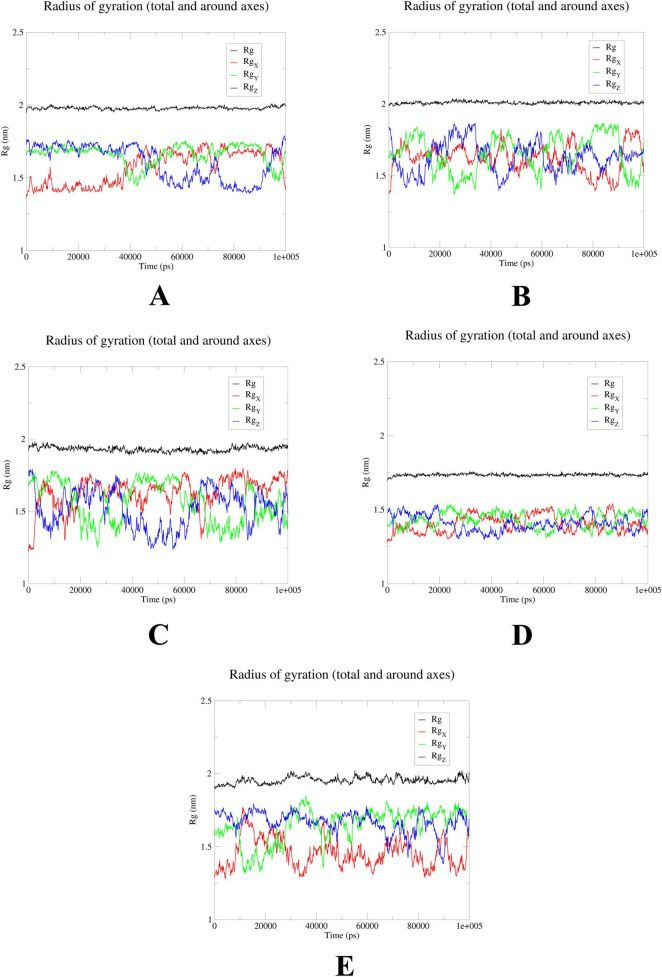
Radius of gyration (Rg). **(A)** AKT1. **(B)** EGFR. **(C)** SRC. **(D)** HSP90AA1. **(E)** PPARG.

**FIGURE 9:**
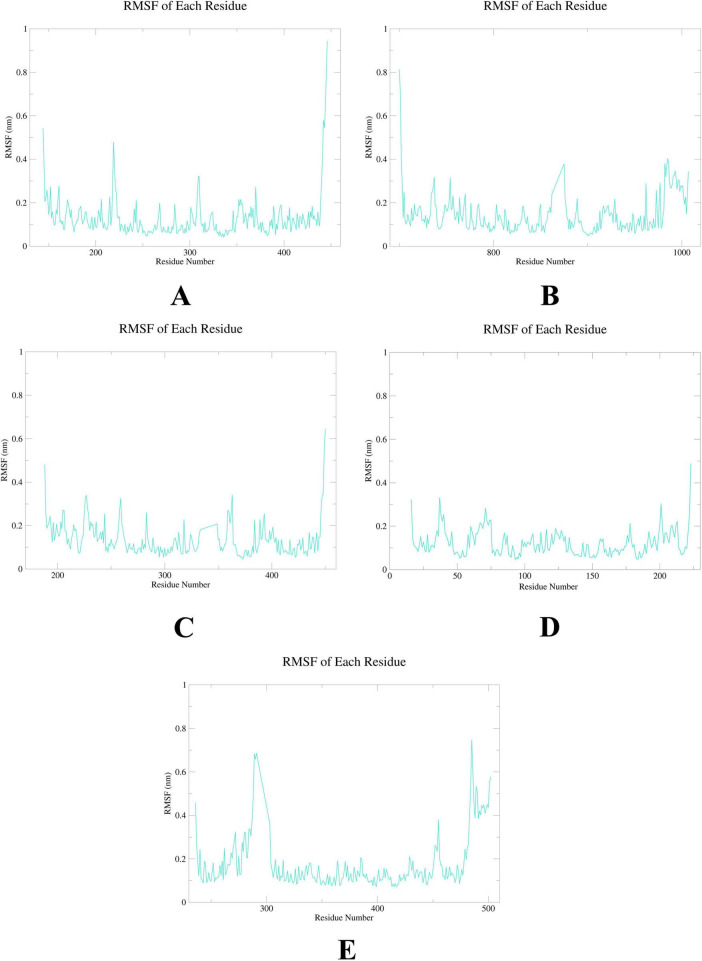
Root mean square fluctuation (RMSF). **(A)** AKT1. **(B)** EGFR. **(C)** SRC. **(D)** HSP90AA1. **(E)** PPARG.

**FIGURE 10:**
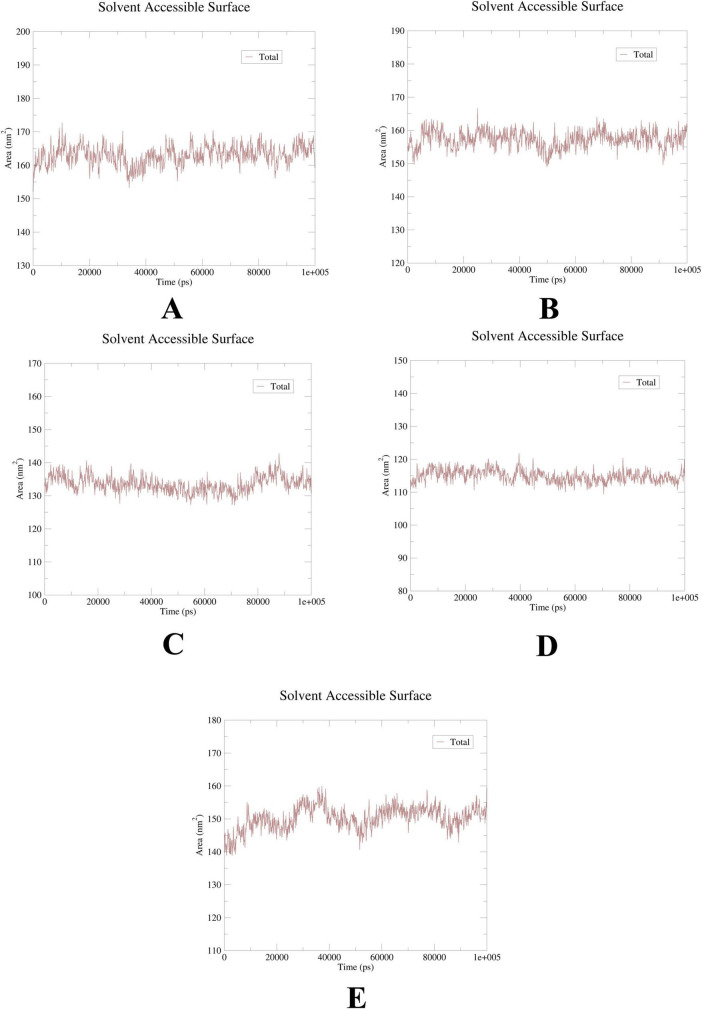
Solution accessible surface area (SASA). **(A)** AKT1. **(B)** EGFR. **(C)** SRC. **(D)** HSP90AA1. **(E)** PPARG.

**FIGURE 11:**
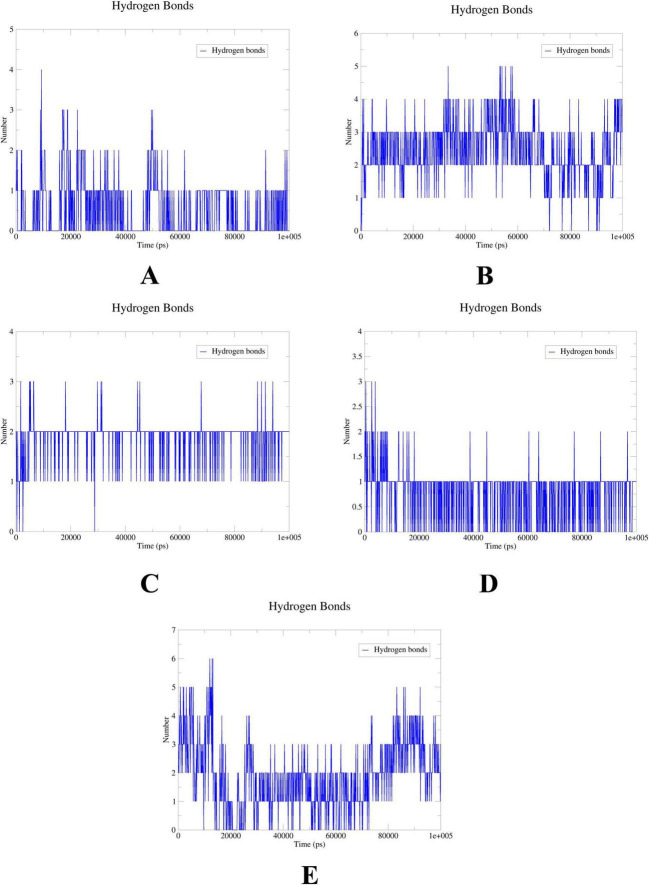
Hydrogen bonds. **(A)** System probenecid-AKT1. **(B)** System probenecid-EGFR. **(C)** System probenecid-SRC. **(D)** System probenecid-HSP90AA1. **(E)** System probenecid-PPAR.

To further elucidate the critical interactions between probenecid and the hub targets, molecular docking models were subjected to molecular dynamics (MD) simulations. First, the root mean square deviation (RMSD) values were analyzed using the gmx trjconv method (Figure 7). The results showed relatively lower and flatter RMSD values for SRC-probenecid, HSP90AA1-probenecid, and EGFR-probenecid, indicating greater stability. In contrast, AKT1-probenecid and PPARG-probenecid exhibited higher and more fluctuating RMSD values. Next, the gyrate (Rg) (Figure 8), root mean square fluctuation (RMSF) (Figure 9), and solvent accessible surface area (SASA) (Figure 10) values for each protein were calculated using the gmx gyrate, RMSF, and SASA methods, respectively. The results revealed that PPARG was more flexible than the other targets, as indicated by its fluctuating Rg and SASA values during the 100 ns simulation process, along with the highest RMSF value. This suggested that the RMSD value of the PPARG-probenecid system could not fully reflect its stability. Additionally, we analyzed hydrogen bond formation in all systems using the gmx hbond method to assess protein-ligand interaction stability (Figure 11). The results showed that probenecid consistently formed hydrogen bonds with SRC, HSP90AA1, and EGFR. Based on these MD simulation results, we speculated that SRC, HSP90AA1, and EGFR are more likely to be the target proteins of probenecid compared to the others.

### Electrostatic potential mapping and SAR results

**FIGURE 12:**
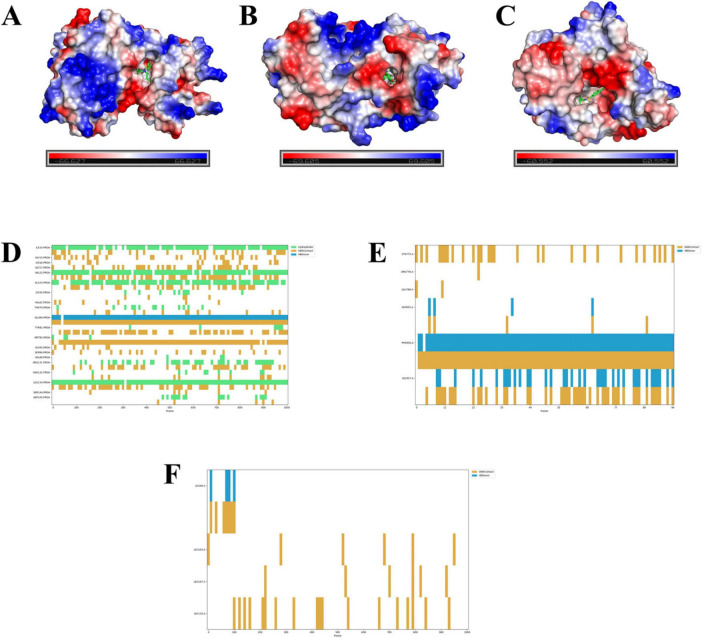
Electrostatic potential and structure activity relationship results.**(A–C)** Electrostatic potential maps of probenecid binding to the A chain of SRC (A), EGFR **(B)**, and HSP90AA1 **(C)**. The color scale represents the electrostatic potential distribution, with red (negative), blue (positive), and white (neutral) indicating the electrostatic potential of the protein surface. The binding sites of probenecid are highlighted in green (ball-and-stick model). **(D–F)** Structure–activity relationship (SAR) results showing the interactions between probenecid and specific amino acid residues in the A chain of SRC **(D)**, EGFR **(E)**, and HSP90AA1 **(F)**. The plots indicate the types of interactions (hydrogen bonding, van der Waals forces, and hydrophobic interactions) and their duration over the simulation period. Key residues involved in binding are labeled, with hydrogen bonds shown in yellow, van der Waals interactions in blue, and hydrophobic interactions in green.

Electrostatic potential mapping reveals that probenecid deeply penetrates the protein cavities of SRC (Figure 12A), EGFR (Figure 12B), and HSP90AA1 (Figure 12C). This suggests that probenecid can interact with specific internal regions of these proteins. Such deep penetration implies that probenecid may bind to the active sites or key domains of these proteins, thereby influencing their functionalities. According to the SAR results, probenecid interacts with the A chain of SRC through hydrophobic interactions and van der Waals forces with residues Ile14, Val22, Ala33, Met82, and Leu134 over a period of time (Figure 12D). Additionally, Glu80 engages in hydrogen bonding and van der Waals interactions with the small molecule. In the A chain of EGFR, Phe856 and Gly857 participate in hydrogen bonding and van der Waals interactions with probenecid over an extended period, highlighting their critical role in protein–small molecule binding (Figure 12E). In contrast, HSP90AA1 is not prioritized as a candidate due to its fewer contacting residues and relative instability (Figure 12F).

## Discussion

SARS-CoV-2/RSV co-infection imposes substantial burdens on public health systems and socioeconomic stability, while directly threatening individual health. Our study investigated the potential of probenecid in treating SARS-CoV-2/RSV co-infection by identifying key molecular targets and pathways through a bioinformatics approach. We determined 141 common targets and pathways associated with both probenecid and SARS-CoV-2/RSV co-infection and proposed probenecid as a potential therapeutic agent based on these findings.

### Probenecid might influence the hub targets to combat SARS-CoV-2/RSV co-infection

A crucial discovery of our study is the identification of 16 hub targets involved in the interaction between probenecid and SARS-CoV-2/RSV co-infection. These key targets—AKT1, ALB, EGFR, CASP3, CTNNB1, SRC, HSP90AA1, MMP9, CXCL8, PPARG, IGF1, ANXA5, MAPK1, BCL2L1, RHOA, and MAPK14—play significant roles in immune regulation, inflammation, and cellular signaling. Importantly, previous studies have reported that several of these targets (AKT1, EGFR, CASP3, MMP9, CXCL8, IGF1, MAPK1, RHOA, and MAPK14) are critical in the therapeutic mechanisms of various infections. However, our findings expand on existing knowledge by elucidating their specific involvement in SARS-CoV-2/RSV co-infection and their potential modulation by probenecid.

AKT1 inhibits autophagy and promotes viral gene expression, including replication (Wang et al., 2014). It is activated in response to SARS-CoV-2 infection and facilitates virus reproduction (Gassen et al., 2021). Simultaneously, RSV infection induces lung epithelial cell death; however, activation of cell survival pathways, including AKT and extracellular signal-related kinase (ERK) pathways, significantly delays cell death (Monick et al., 2004). EGFR, a tyrosine kinase, triggers inflammation and mucin production (Kalinowski et al., 2018). During the early stages of COVID-19 pathogenesis, EGFR is down-regulated, contributing to the progression and spread of the viral infection (Turk et al., 2020). In RSV infection, EGFR is activated in lung epithelial cells, promoting the pro-inflammatory response (Currier et al., 2016). However, inhibiting EGFR may enhance the innate immune response of respiratory epithelial cells to RSV, potentially hindering viral clearance (Kalinowski et al., 2014). CASP3, a protease crucial for mediating apoptosis, plays a role in maintaining cellular homeostasis (Chen et al., 2022). Activation of CASP3 serves as a defense mechanism against SARS-CoV-2-induced damage (Yildiz et al., 2022). Similarly, CASP3 activation delays RSV infection (Niyomdecha et al., 2021). MMP9, a zinc-binding endopeptidase, degrade various extracellular matrix molecules (Yabluchanskiy et al., 2013) and is associated with the development of respiratory diseases, including cystic fibrosis and acute lung injury (ALI) (Devereux et al., 2014). In SARS-CoV-2 infection, MMP9 contributes to inflammation, particularly during the recovery phase (Gelzo et al., 2022). Notably, MMP9 levels have been identified as potential pathophysiologic and prognostic markers for predicting in-hospital mortality (D Avila-Mesquita et al., 2021). In RSV infection, MMP9 mediates neutrophil recruitment and viral clearance (Dabo et al., 2015).

CXCL8, a pro-inflammatory chemokine, serves as a primary chemoattractant for neutrophils (Espindola et al., 2021). Elevated levels of plasma CXCL8 have been associated with increased mortality in patients with COVID-19 (Pius-Sadowska et al., 2022). Additionally, elevated levels of CXCL8 in cerebrospinal fluid (CSF) have been linked to para-infectious central nervous system (CNS) inflammatory symptoms caused by SARS-CoV-2 infection (Espindola et al., 2021). CXCL8 has also been found in abundance within the lung tissues of patients infected with respiratory syncytial virus (RSV), and its levels correlate with the severity of RSV infection (McNamara et al., 2005; Smyth et al., 2002). IGF1 is a growth factor that enhances vascular protection, promotes tissue repair, and reduces pro-inflammatory cytokines (Munoz et al., 2021). In patients with COVID-19, the IGF1 levels are elevated during the early stages of infection (Zhao et al., 2021). Furthermore, individuals with low serum IGF1 levels are at a higher risk of developing COVID-19 compared to those with normal serum levels (Fan et al., 2021). IGF1R, the receptor for IGF1, is also one of the entry receptors for RSV (Griffiths et al., 2020). MAPKs are associated with multiple cellular functions, including differentiation, division, and death (Monick et al., 2001). They play a critical role in inflammation induced by SARS-CoV-2 infection (Galganska et al., 2021). In patients with severe COVID-19, hyperoxia and mechanical ventilation can exacerbate lung injury, which can be mitigated by inhibiting MAPK activity (Parinandi et al., 2003). Moreover, exposure to RSV has also been shown to upregulate MAPK14 expression (Choudhary et al., 2005). The small GTP-binding protein RHOA, a member of the Ras superfamily (Gower et al., 2005), is activated during RSV infection (Gower et al., 2001). As a GTPase, RHOA interacts with the RSV fusion protein to promote virus-induced syncytium formation (Pastey et al., 2000). Recently, RHOA has been identified as one of the key hub genes involved in the immunopathogenesis of COVID-19 (Hasankhani et al., 2021).

### The therapeutic mechanisms for probenecid to anti-SARS-CoV-2/RSV co-infection

The results of GO and KEGG pathway analyses further suggested that probenecid exerts its therapeutic effects by modulating immune responses, inflammatory processes, immune cell differentiation, and signal transduction through key pathways. These pathways include TLRs, NLRs, NF-κB, and JAK-STAT signaling pathways, as well as cytokine-cytokine receptor interactions and apoptosis regulation. These findings highlight the potential of probenecid in mitigating the excessive inflammatory response and immune dysregulation observed in SARS-CoV-2/RSV co-infection.

TLRs are a class of pattern recognition receptors (PRRs) capable of identifying pathogen-associated molecular patterns (PAMPs). This recognition activates the body’s innate immune response against pathogen invasion through various signaling pathways. Multiple TLR signaling pathways can be activated by viruses. For instance, TLR2 recognizes the spike glycoprotein of SARS-CoV-2, while TLR3, TLR7, and TLR8 recognize its RNA (Ciaston et al., 2022). Similarly, TLRs recognize the RSV F protein. Specifically, TLR4 mediates IL-6 production by monocytes in response to the RSV F protein, thereby inhibiting RSV replication *in vivo* (Shang et al., 2021). Like NLRs, the NOD-like receptor family pyrin containing 3 (NLRP3) inflammasome can be activated by viral proteins such as ORF3a in COVID-19. This activation leads to abnormal IL-1β secretion and severe inflammation (Bertoni et al., 2022; Sun et al., 2022). The RSV small hydrophobic (SH) protein also mediates IL-1β secretion by activating the NLRP3 inflammasome, inducing inflammatory responses. However, inhibiting the NLRP3 inflammasome can prevent the development of pulmonary immunopathology and long-term airway disease caused by RSV infection (Malinczak et al., 2021). The NF-κB pathway is stimulated by the SARS-CoV-2 N protein, inducing acute lung injury in mice (Xia et al., 2021). By enhancing the expression of inflammatory cytokines, chemokines, alarmins, and inducible enzymes, SARS-CoV-2-mediated NF-κB activation triggers a cytokine storm (Attiq et al., 2021). Previous studies have shown that RSV can activate NLRP3 inflammasomes through the NF-κB pathway. Additionally, the activation of NF-κB target genes, such as pro-inflammatory cytokines IL-1β, leads to an inflammatory response (Shen et al., 2019). Research has demonstrated that inhibiting the JAK-STAT signaling pathway can regulate COVID-19-associated cytokine release syndrome (Goker and Biray, 2020; Luo et al., 2020). Recent reports have also linked more severe RSV infections to higher JAK-STAT3 pathway activity (Bouwman et al., 2020).

Acute respiratory distress syndrome (ARDS) and several organ dysfunction syndromes associated with COVID-19 are primarily induced by cytokine storms. Multiple studies have confirmed a positive correlation between the severity of COVID-19 and the levels of C-reactive protein (CRP), IL-2, IL-6, IL-7, IL-8, and TNF-α cytokines (Huang et al., 2020; Zeng et al., 2020). Similarly, during the natural immunological phase of anti-RSV infection, RSV binds to TLRs on the surface of epithelial cells, enhancing the expression of pro-inflammatory cytokines such as IL-6 and TNF-α, thereby triggering an inflammatory response (Langedijk and Bont, 2023). Additionally, RSV can induce the release of IL-4, IL-5, IL-9, IL-10, and IL-13 from Th2 cells, initiating a strong Th2 cytokine response and inhibiting the development of Th1 cells, leading to Th1/Th2 immunological imbalance (Openshaw and Chiu, 2013). TNF, a potent cytokine, is crucial for the induction and regulation of inflammatory immune responses (Shivappa et al., 2017). Following coronavirus infection, TNF and TNF receptor 2 signaling pathways promote leukocyte recruitment, activation, and host cell survival (Cheng et al., 2021). Moreover, RSV promotes TNF synthesis through its replicative activity in macrophages and activates necroptosis even when macrophages are destined for cell death. Consequently, TNF release via necroptosis activation creates a positive feedback loop, exacerbating RSV-induced lung disease (Santos et al., 2021). According to available data, apoptosis is critical for SARS-CoV-2 infection. In the early phase, SARS-CoV-2 inhibits apoptosis to evade elimination and secure time and space for replication. Later, the virus significantly promotes apoptosis in host cells, causing severe tissue damage and functional loss, thereby accelerating disease progression and potentially leading to death (Li X. et al., 2022). Similarly, RSV induces autophagy-mediated inhibition of apoptosis to facilitate its replication (Li et al., 2018), and RSV infection of the central nervous system (CNS) may lead to apoptosis in Neuro-2a (N2a) neuronal cells, resulting in neurological symptoms (Yuan et al., 2018).

To further validate our findings, we conducted molecular docking, MD simulations, electrostatic potential mapping and SAR to explore the binding interactions between probenecid and the identified core targets. Molecular docking and MD simulations revealed that probenecid exhibits strong binding affinities with AKT1, EGFR, SRC, HSP90AA1, and PPARG, with stable interactions observed for SRC, EGFR, and HSP90AA1, suggesting their potential roles in mediating its therapeutic effects. Electrostatic potential mapping further showed that probenecid deeply penetrates the protein cavities of SRC, EGFR, and HSP90AA1, possibly binding to their active sites or key functional domains to modulate their biological activity. SAR analysis indicated that key residues in SRC and EGFR play crucial roles in hydrogen bonding, hydrophobic interactions, and van der Waals forces, whereas HSP90AA1 exhibited weaker interactions due to fewer contacting residues and relative instability. Therefore, SRC and EGFR may serve as key targets for probenecid in treating SARS-CoV-2/RSV co-infection, while HSP90AA1 may play a lesser role. These findings provide new molecular insights into probenecid’s drug repurposing potential and lay the foundation for further experimental validation.

In conclusion, our study not only identifies critical molecular targets and pathways through which probenecid may exert its therapeutic effects but also provides mechanistic insights into its mode of action. By integrating bioinformatics analyses, molecular docking, MD simulations, electrostatic potential mapping and SAR, we present strong evidence supporting the potential of probenecid as a novel therapeutic strategy for SARS-CoV-2/RSV co-infection. Future experimental validation of these findings will be essential to confirm its clinical applicability.

## Conclusion

Collectively, our findings from systems pharmacology and bioinformatics analysis indicate that immune and inflammatory responses play a pivotal role in the therapeutic effects of probenecid. Infectious disease-related pathways also contribute significantly to its effectiveness in treating SARS-CoV-2/RSV co-infection. Further validation was conducted through molecular docking, MD simulations, electrostatic potential mapping, and SAR analysis. These analyses suggest that SRC and HSP90AA1 are the potential binding targets of probenecid. This study provides valuable preliminary insights into the molecular mechanisms of probenecid’s action. It establishes a strong foundation for future research to explore its potential as a therapeutic strategy for SARS-CoV-2/RSV co-infection.

## Data Availability

The original contributions presented in this study are included in this article/Supplementary material, further inquiries can be directed to the corresponding authors.

## Footnotes

1
https://covid19.who.int/

2
http://www.swisstargetprediction.ch/

3
http://www.stitch.embl.de/

4
http://www.lilab-ecust.cn/pharmmapper/

5
https://string-db.org/

6
http://ctdbase.org/

7
https://www.disgenet.org/covid/diseases/summary/

8
https://rgd.mcw.edu/wg/portals/

9
https://www.genecards.org/

10
https://www.disgenet.org

11
https://omim.org/

12
http://jvenn.toulouse.inra.fr/app/example.html

13
https://www.rcsb.org/
